# Cerebrovascular pulsatility indicates preoperative subcortical cognitive impairment and an increased risk for postoperative delirium in elderly patients undergoing elective spine surgery

**DOI:** 10.3389/fmed.2024.1433380

**Published:** 2024-09-24

**Authors:** Angelika Saar, Jonas Müller, Yannick Veser, Frederik Behr, Eiko Rathmann, Henry W. S. Schroeder, Agnes Flöel, Jan-Uwe Müller, Bettina von Sarnowski, Robert Fleischmann

**Affiliations:** ^1^Department of Neurology, University Medicine Greifswald, Greifswald, Germany; ^2^Department of Psychosomatics and Psychotherapy, Bethanien Hospital for Psychiatry, Greifswald, Germany; ^3^Department of Neurosurgery, University Medicine Greifswald, Greifswald, Germany; ^4^Institute of Neuroradiology, University Medicine Greifswald, Greifswald, Germany

**Keywords:** delirium, carotid ultrasound, cognitive impairment, white matter lesions, risk prediction, spine surgery

## Abstract

**Introduction:**

Advances in spine surgery enable safe interventions in elderly patients, but perioperative neurocognitive disorders (pNCD), such as post-operative delirium (POD) and cognitive dysfunction (POCD), remain a serious concern. Pre-operative cognitive impairment is a major risk factor for pNCD. Comprehensive pre-operative cognitive assessments are not feasible in clinical practice, making effective screening methods desirable. This study investigates whether pre-operative cerebrovascular duplex sonography can assess subcortical (vascular) cognitive impairment and the risk for POD.

**Methods:**

This prospective single-center study recruited patients aged ≥60 years scheduled for elective spine surgery at a German university hospital. Patients underwent pre-operative assessments including cognitive abilities (CERAD test battery), structural MRI, and cerebrovascular duplex sonography. POD screening was conducted three times daily for at least 3 days. The primary hypothesis, that the mean pulsatility index (PI) of both internal carotid arteries (ICA) predicts POD risk, was tested using logistic regression. Secondary analyses examined the association between POD risk and ICA flow (time-averaged peak velocities, TAPV) and correlations with cognitive profiles and MRI characteristics.

**Results:**

POD occurred in 22% of patients (*n* = 22/99) within three postoperative days. Patients with POD were significantly older (75.9 ± 5.4 vs. 70.0 ± 6.9 years, *p* < 0.01) but did not differ by gender (*p* = 0.51). ICA PI significantly predicted POD risk (OR = 5.46 [95%CI: 1.81–16.49], *p* = 0.003), which remained significant after adjustment for age and duration of surgery (OR_adj_ = 6.38 [95% CI: 1.77–23.03], *p* = 0.005). TAPV did not inform the POD risk (*p* = 0.68). ICA PI Pre-operative cognitive scores were significantly associated with ICA PI (mean CERAD score: *r* = −0.32, *p* < 0.001). ICA PI was also significantly associated with total white matter lesion volume (*τ* = 0.19, *p* = 0.012) and periventricular white matter lesion volume (*τ* = 0.21, *p* = 0.007).

**Discussion:**

This is the first study to demonstrate that cerebrovascular duplex sonography can assess the risk for POD in elderly spine surgery patients. Increased ICA PI may indicate subcortical impairment, larger white matter lesion load, and lower white matter volume, predisposing factors for POD. Pre-operative cerebrovascular duplex sonography of the ICA is widely available, easy-to-use, and efficient, offering a promising screening method for POD risk. Increased ICA PI could supplement established predictors like age to adjust surgical and peri-operative procedures to individual risk profiles.

## 1 Introduction

Advances in spine surgery have significantly improved the safety and efficacy of interventions, particularly among elderly patients suffering from disabling spine diseases (1). Despite these advancements, perioperative neurocognitive disorders (pNCD), importantly postoperative delirium (POD), remain a serious and prevalent concern since their occurrence is associated with inferior outcomes (2). POD is characterized by acute onset and fluctuating levels of consciousness, and impaired attention. POD may not be transient but about one third of affected patients suffer post-operative cognitive dysfunction (POCD) (3). There is notable evidence for non-pharmacological, multicomponent strategies to prevent POD and its sequelae, adaptable to various settings (4). Further measures to reduce the risk of POD following spine surgery are to adapt interventions to the risk profile, e.g., limiting the duration and extent of surgery (5). Allocating resources and tailoring interventions to patients at the highest risk of developing POD could thus enhance outcomes and optimize the use of healthcare resources. Unfortunately, identification of patients of risk for pNCD is challenging. Unidentified preoperative cognitive impairment was recently identified as a major gap in patients undergoing elective surgery (6). Comprehensive cognitive assessments cannot mitigate this challenge since these are time-consuming, resource-intensive, and requires trained personnel (7).

The use of technology in clinical settings can significantly enhance the efficiency by facilitating the rapid and effective assessment of large patient populations. Cerebrovascular duplex sonography (CVDS) is a non-invasive imaging technique that can provide insights into cerebrovascular brain health (8). It is acknowledged that CVDS provides valuable indirect information about cerebral small vessel disease (SVD) (9). Subcortical vascular cognitive impairment (VCI), a consequence of SVD, often precedes severe cognitive decline and serves as an early indicator of delirium risk (10, 11). CVDS may provide well-suited surrogate markers associated with VCI because it provides a non-invasive assessment of cerebral hemodynamics. The pulsatility index (PI) measured by duplex sonography of the ICA reflects cerebrovascular resistance and has been associated with white matter lesions and brain atrophy, which are key indicators of VCI (12). Additionally, the time-averaged peak systolic velocity (TAPV) serves as a surrogate marker for brain atrophy, with lower TAPV values indicating reduced cerebral perfusion, linked to neurodegenerative processes and cognitive decline (13).

This study investigates the primary hypothesis that CVDS-detected changes in cerebral small vessel disease (SVD) can predict the risk of POD. The secondary hypothesis under investigation is that this risk prediction is linked to a correlation between CVDS measures and vascular cognitive impairment (VCI). Additionally, we explore whether CVDS changes are associated with indicators of SVD on cerebral magnetic resonance imaging (MRI), thereby supporting its utility as a surrogate marker for VCI. To test these hypotheses, patients undergoing elective spine surgery will receive multimodal pre-operative assessments, including CVDS, multi-domain neurocognitive tests, and neuroimaging. Since VCI accounts for up to 39% of cognitive impairment cases (14), confirming CVDS’s predictive value for pre-operative VCI and POD risk would support its use in tailored peri-operative management strategies.

## 2 Methods

### 2.1 Study registration and data availability

This prospective single-center study was prospectively registered at clinicaltrials.gov (NCT03486288), approved by the Institutional Ethics Review Board of the University Medicine Greifswald (BB 192/17) and adhered to the standards of the Helsinki Declaration in its latest revision. Data that support the findings of this study are available from the corresponding author upon reasonable request.

### 2.2 Study design and patient population

The evaluation of CVDS for the prediction of VCI and presence of small vessel disease was predefined as secondary endpoint in the *Cognitive Dysfunction Following Elective Spine Surgery in Elderly Patients* (CONFESS) study. A comprehensive overview of the study methodology is provided in the study protocol (8). The primary endpoint of this study was the validation of duration of surgery as a modifiable risk factor for the occurrence of POD, which was confirmed and previously reported (5). The CONFESS study was jointly conducted by the departments of Neurology and Neurosurgery at a tertiary care hospital. Patients scheduled for elective spine surgery were consecutively recruited through the outpatient clinic of the department of Neurosurgery over a period of 2 years. Inclusion criteria were age ≥60 years, elective spine surgery without opening the dura, ability to provide informed consent themselves, and German native speakers. Exclusion criteria were any known diagnosis of dementia or neurodegenerative disease, psychiatric disease, prescription of central nervous system–active medication (e.g., antidepressants, antipsychotics, sedatives, alpha-1-receptor antagonists), inability to participate in follow-up and presence of electronic or displaceable metallic implants that preclude magnetic resonance imaging (MRI). Anesthesia was induced with intravenous sufentanyl (0.3–0.6 mg/kg) and propofol (1.5–2.5 mg/kg), with muscle relaxation achieved using cisatracurium (1.5 mg/kg). Anesthetic depth was monitored using bispectral index (BIS) and real-time electroencephalography (EEG), ensuring the target range for sevoflurane was maintained at 0.8–1.0 MAC. Vital parameters were continuously monitored, with arterial lines placed in select patients based on their preoperative risk profiles.

Pre-operative baseline assessments were done 7 ± 7 days prior to surgery (V0). These included evaluations of demographic data, functional abilities (Oswestry disability index, ODI; Barthel Index, BI), medical history, comprehensive neurocognitive tests and structural MRI. The ODI ranges from 0% (no disability) to 100% (worst disability). The BI ranges from 0 (maximum dependence) to 100 points (completely independent). Screening for POD was done immediately post-operatively (V1), and then three-times daily for at least 72 h, or until POD resolved (V2). The decision to screen for POD for a minimum of 72 h is grounded in the understanding that it typically develops within the first 3 days after surgery while cases of delirium occurring later are more likely to indicate postoperative complications, such as infections, which were not the focus of this study (15). Patients were reassessed 3 months post-operatively including identical examinations of neurocognitive abilities and structural MRI (V3).

### 2.3 Cerebrovascular duplex sonography

The patient was positioned supine with the head extended and rotated opposite to the side of the artery being examined. All examinations were done by an experienced sonographer. An ultrasound transducer operating at 9 Mhz was used for extracranial duplex sonography of the internal carotid artery (ICA), common carotid artery (CCA) and vertebral artery (VA). Transcranial duplex sonography of the middle cerebral artery (MCA), anterior cerebral artery (ACA) and posterior cerebral artery (PCA) was done using low-frequency ultrasound operating at 2 Mhz. Clear visualization of arteries was required and cases permitting only a projection to vessel parts were excluded. The same transducer was used for transforaminal insonation of the intradural segments of the VA and the basilar artery (BA). B-mode ultrasound was employed extracranially to visualize the anatomical structure of the carotid arteries, with particular focus on the bifurcation and the proximal internal carotid artery. The angle of insonation was adjusted to remain below 60 degrees for accurate velocity measurement. The Doppler gate was positioned at the center of the artery to capture the laminar flow, avoiding the vessel wall and plaque areas which might distort the waveform. An envelope of the ICA spectral waveform was generated by the ultrasound machine to demarcate the peak systolic and end-diastolic velocities. Using these data points, the machine calculates the Time-Averaged Peak Velocity (TAPV) and the Pulsatility Index (PI). TAPV is derived from the mean of peak systolic velocities across multiple cardiac cycles, providing an indication of the maximum velocity achieved by blood flow. Changes of ICA TAPV values were shown to correlate with brain atrophy and cerebral small vessel disease (16). The Pulsatility Index (PI) was computed using the formula: (Peak Systolic Velocity − End Diastolic Velocity)/Mean Velocity. This index is a surrogate marker for vascular resistance distal to the sensor location. Elevated PI values are typically indicative of increased downstream vascular resistance, e.g., in cerebral small vessel disease or (subcortical) brain atrophy. This being said, TAPV and PI reflect different flow parameters, both of which may inform the presence of intracranial vascular and structural pathology. Automated calculations enhance the reproducibility and accuracy of these measurements, essential for longitudinal monitoring and comparative analysis. The mean TAPV and PI of both ICA were considered for statistical evaluations. Beyond quantitative analyses of the ICA spectral waveform envelope, extra-and intracranial cerebral vasculature was assessed for local pathology and hemodynamic stenoses as recommended (17, 18). Pathologies of ICA hemodynamics were rated according to *North American Symptomatic Carotid Endarterectomy Trial* (NASCET) criteria (19). Changes in other arteries were rated as mild, moderate, severe or occluded by the investigator.

### 2.4 Neurocognitive assessment

The CERAD-NP Plus (Consortium to Establish a Registry for Alzheimer’s Disease—Neuropsychological) test battery was used for the comprehensive assessment of cognitive abilities (20). The test battery typically takes about 45–90 min to complete. Each test is scored according to standardized protocols, and the scores are interpreted in relation to normative data to identify cognitive impairment. The test battery includes multiple tests. Verbal Fluency assesses executive function and language. The Modified Boston Naming Test evaluates language function, with patients naming a series of line drawings. The Mini-Mental State Examination (MMSE) screens for general cognitive impairment, covering orientation, registration, attention and calculation, recall, language, and visuospatial skills. The Word List Memory test assesses episodic memory, where patients learn a list of words and recall them immediately and after a delay. Constructional Praxis evaluates visuospatial and constructional abilities, with patients copying geometric figures. Word List Recall evaluates delayed recall ability, asking patients to recall words learned earlier. Word List Recognition tests recognition memory, where patients identify learned words from a list that includes distractors. Constructional Praxis Recall assesses visual memory, with patients redrawing geometric figures from memory after a delay. The Trail Making Test assesses processing speed and executive function. The cognitive domains tested include memory, language, executive function, visuospatial and constructional abilities, and general cognitive function. Cortical cognitive impairment is indicated by deficits in memory, language, and visuospatial/constructional abilities. Specifically, the tests for cortical impairment include Word List Memory (immediate and delayed recall), Word List Recognition, Constructional Praxis Recall, Modified Boston Naming Test, Verbal Fluency, and Constructional Praxis. Subcortical cognitive impairment is indicated by deficits in executive function, attention, and processing speed. The tests relevant for subcortical impairment include Verbal Fluency, the Trail Making Test, and components of the Mini-Mental State Examination (MMSE). Individual test scores and their mean score across domains were *z*-transformed to provide age, gender and education adjusted values (21).

### 2.5 Magnetic resonance imaging

MR images were acquired at the Baltic Imaging Center (Center for Diagnostic Radiology and Neuroradiology, University Medicine Greifswald) on a 3-T Siemens Verio scanner using a 32-channel head coil. Structural MRI scans included a high resolution T1-weighted structural image acquired using Magnetization-Prepared Rapid Gradient-Echo (MP-RAGE, 1 mm^3^ isotropic voxel, TR = 2,300 ms, TE = 2.96 ms, matrix) sequence to provide anatomical reference. Furthermore, isometric T2-weighted fluid-attenuated inversion recovery (FLAIR) images were acquired (slice thickness 1 mm, TR/TE 7000/379, matrix 256). Preprocessing included functional realignment, slice-time correction, structural segmentation and normalization to the Montreal Neurological Institute (MNI) template, functional segmentation and normalization, and smoothing (with 6 mm Gaussian kernel). Segmentation of the structural images was carried out using CAT12 Toolbox (v.12.8r1932; Christian Gaser, Jena University Hospital), yielding gray matter, white matter and cerebro-spinal fluid masks in the MNI template space. Calculations included total intracranial, grey matter and white matter volume. Furthermore, the volume of white matter lesions was calculated as absolute volume (cm^3^) and normalized to the total intracranial volume (%).

### 2.6 Statistical analysis

Data evaluation was done using IBM SPSS Statistics (v29, Amonk, New York, United States). Results from descriptive statistics are reported as mean ± SD or median and interquartile range depending on data distribution. Results from inferential statistics are reported with their appropriate coefficients and, if applicable, odds ratios (OR) including 95% CI in brackets and *p*-values denoting the statistical significance. The primary hypothesis of this study was that POD occurrence is predicted by quantitative ultrasound parameters, i.e., PI and TAPV. Given that PI is more closely tied to increased remote resistance related to small-vessel disease (22), this was chosen as primary endpoint to predict delirium occurrence. Delirium occurrence was defined as binary dependent variable, which was either positive in case of any episode of delirium in the screening period or otherwise negative. The hypothesis was tested using binary logistic regression with PI mean as predictor and including a constant. We subsequently tested whether the model explain POD can be further improved by including cognitive test results and imaging characteristics, as well as age and surgery duration which are known to influence the risk of POD (5). This approach allowed us to determine whether the mean PI is an independent predictor of POD. The model was optimized using a backward stepwise method, with the Akaike Information Criterion (AIC) employed to assess the goodness of fit. Only models with an AIC superior to the starting model, which included only the constant and mean PI, were selected for further consideration. The predictive value of TAPV was tested using the same statistical model as secondary endpoint. Diagnostic properties of PI and TAPV were assessed using receiver operating characteristics (ROC) analyses. Stenoses of the cerebral vasculature were considered as predictors of POD occurrence with an ordinal scale (none, mild, moderate, severe, occlusion) and appropriate linking function in a generalized linear model (GLM). The correlation between ultrasound parameters and cognitive performance in the CERAD mean score and its subtests was tested using Pearson correlation adjusted for alpha error accumulation using false discovery rate (FDR) corrections (23). Comparisons of ultrasound and neuroimaging findings were done using Kendall’s rank correlation given that the distribution of neuroimaging findings was non-normal. An alpha error of 5% is considered as level of significance in two-tailed test throughout inferential statistics. *p*-values are rounded to three decimal places and values lower than 0.001 are not reported exact but as <0.001.

## 3 Results

### 3.1 Patient description and delirium occurrence

We screened 565 patients for eligibility, 124 eligible patients consented to participate, and 99 patients (age: 71.3 ± 7.0 years, 49 female) completed baseline examinations and the postoperative in-hospital period (V0–V2; others withdrew their consent or intervention was cancelled). Anesthesiologic and surgical characteristics were as follows. Most of the patients received lumbar fusion and lumbar decompression of 1.8 ± 1.2 spinal levels, which took on average 192.7 ± 110.7 min. The ODI was 40.7 ± 16.3% and the BI 92.4 ± 15.9 points. The level of education was primary school (20%), Junior high school (50%) and high school or higher in 16% of the patients; 9% of patients provided no answer and 5% did not complete school.

POD occurred in 22% of patients (*n* = 22/99). Patients with POD were significantly older (75.9 ± 5.4 vs. 70.0 ± 6.9 years, *p* < 0.01) but did not differ with respect to gender (*p* = 0.51). Patient with POD furthermore underwent significantly longer surgery (247 ± 120 vs. 176 ± 103 min, *p* < 0.001) while gender, educational status, prior amount of alcohol consumption or smoking, type of surgery, surgical complexity, intraoperative fluid and blood turnover, blood oxygenation, temperature or heart frequency were not different between patients with or without POD (all *p* > 0.05).

### 3.2 CVDS findings and prediction of POD

**Figure 1:**
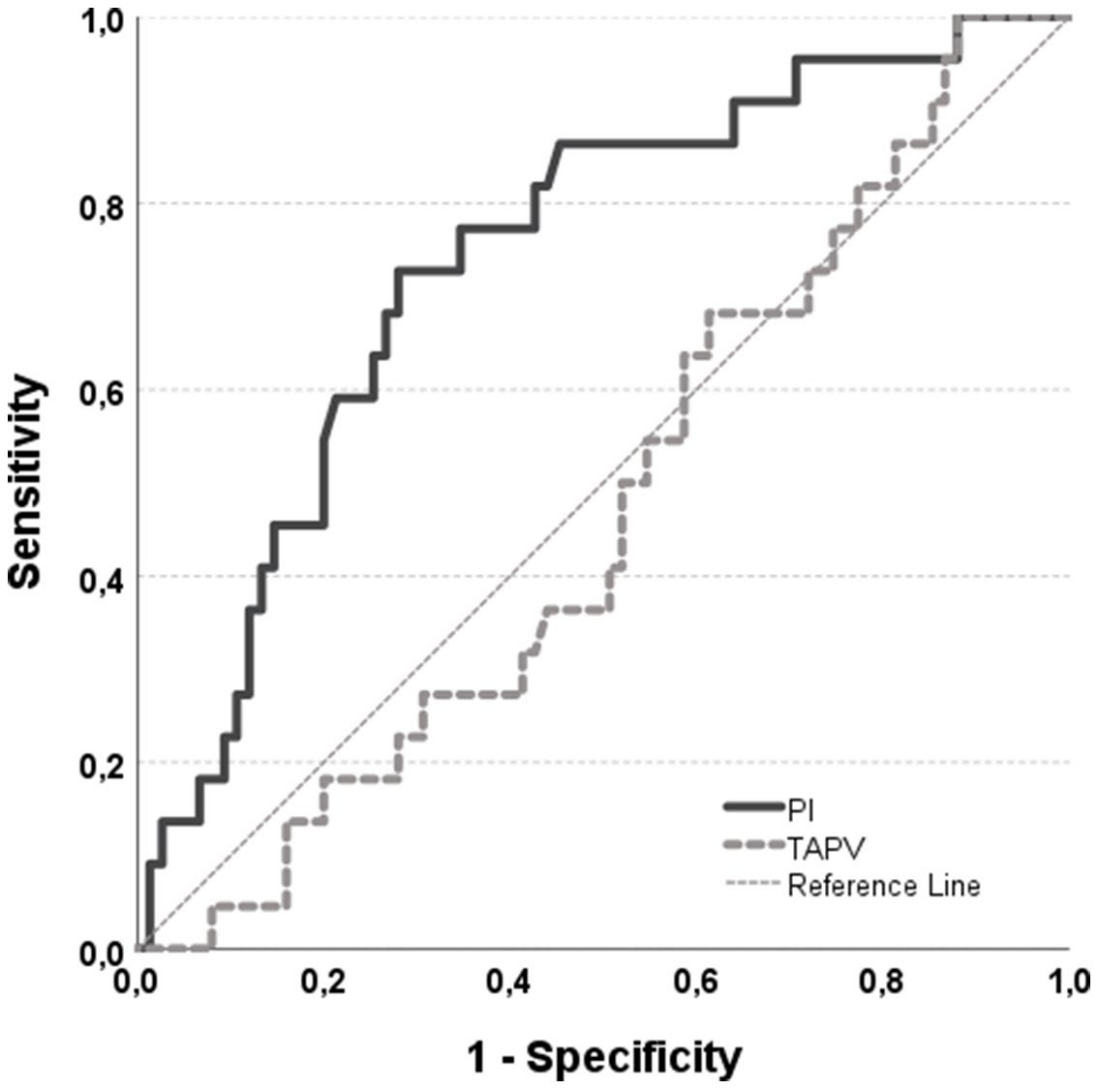
ROC curve illustrating the diagnostic performance of CVDS measures in predicting POD. The data indicate that the PI, in contrast to TAPV, is effective in predicting the occurrence of POD. The diagnostic accuracy is moderate, with a sensitivity of 73% and a specificity of 72% at a PI threshold of 1.68. The AUC for the PI ROC curve is 0.74, with a Gini index of 44%. Given the high prevalence, PI is more effective at identifying patients with a lower rather than a higher risk of developing POD.

All patients were amenable to CVDS investigations pre-operatively, and investigations of extracranial arteries were possible without exception. Transcranial ultrasound was impeded by an insufficient transcranial bone window in 48 patients, i.e., 51 patients received examinations of intracranial arteries. Pathological findings of hemodynamic profiles were rare. There were 6 patients with a 50% stenosis of one of the ICA, 2 patients had a 60% stenosis, and 1 patient had a 70% stenosis (all based on NASCET criteria). All stenoses were unrelated to any clinical event, i.e., they were rated asymptomatic. Incident hemodynamic stenoses in other vessels were mild stenoses in the left (*n* = 1) and right (*n* = 2) MCA, and right PCA (*n* = 1). Other examinations were unremarkable expect for mild arteriosclerotic changes with hemodynamic consequences. Given the small case numbers of incident stenoses, no inferential statistics were applied. Results for PI (1.53 ± 0.74) and TAPV (37.5 ± 17.57) were available in all patients. ICA PI significantly differed between patients with (1.79 ± 0.47) and without POD (1.37 ± 0.52). An increase of the PI by one point was associated with an about 5-fold increased risk for POD (OR = 5.46 [95%CI: 1.81–16.49]). ROC Analyses were performed to assess the diagnostic accuracy of these changes. The Area Under the Curve (AUC) was 0.74 for the PI (*p* < 0.001) and 0.47 for the TAPV (*p* = 0.71, Figure 1). When using a PI of 1.68 as cut-off, the sensitivity and specificity for detecting patients with subsequent POD were 73 and 72%, respectively. The positive and negative predictive values are about 43 and 90%, respectively. There was no significant association between the TAPV and POD status (*p* = 0.68).

### 3.3 Association of CVDS with pre-operative neurocognitive performance

Pearson correlation coefficients were calculated to estimate the association between PI and pre-operative cognitive impairment. There was a significant moderate correlation of the PI with the mean CERAD Score (*r* = −0.32, *p* < 0.001). Results for single subtests of the CERAD are summarized in Table 1. In summary, cognitive tests associated with subcortical impairment were affected as indicated by significant negative correlations with the MMSE while TMT-B was only marginally significant. A negative correlation between word list recognition and Boston naming test furthermore indicate a significant association with inferior memory function.

**Table 1:** Summary of correlation Pearson correlation coefficients between mean PI and CERAD subtests.

CERAD instrument	*z*-score and standard deviation	Correlation coefficient	*p*-value_adj_
Verbal fluency	−0.08 ± 1.21	−0.16	0.11
Boston Naming Test*	0.33 ± 1.16	−0.25	0.03
Mini Mental State Exam*	−0.92 ± 1.98	−0.40	<0.001
WLL learning*	−0.61 ± 1.33	−0.29	0.03
WLL recall	−0.01 ± 1.66	−0.17	0.11
WLL recognition*	−0.38 ± 1.26	−0.23	0.05
Visuo-construction copy	−0.17 ± 1.53	−0.17	0.11
Visuo-construction recall	−0.25 ± 1.64	−0.21	0.10
Trail Making Test A	−0.27 ± 1.18	−0.18	0.11
Trail Making Test B	0.22 ± 1.14	−0.23	0.06
Phonemic fluency	0.16 ± 1.37	0.12	0.25
Total Score*	−0.18 ± 0.91	−0.32	<0.001

* indicates results are statistically significant adjusted for false-discovery rate.

### 3.4 Association of CVDS with neuroimaging results

Seventy-nine patients underwent pre-operative MRI (age: 71.4 ± 7.0 years, 40 female). There was no statistically significant difference of this subgroup in comparison to the complete patient sample (age: *p* = 0.54, gender: *p* = 0.93). Same applied to CERAD results (all *p* > 0.05). Bivariate correlation analyses were conducted to examine the relationship between CVDS and neuroimaging findings. Results are summarized in Table 2.

**Table 2:** Summary of correlation Pearson correlation coefficients between mean PI and CERAD subtests.

MRI parameter	PI		TAPV	
	Correlation coefficient	*p*-value_adj_	Correlation coefficient	*p*-value_adj_
White matter volume	−0.14	0.11	0.20	0.14
Grey matter volume	−0.02	0.95	0.09	0.39
Cerebrospinal fluid volume	0.16	0.11	0.02	0.86
Total lesion volume*	0.19	0.04	−0.15	0.39
Periventricular lesion volume*	0.21	0.04	−0.16	0.39
Juxtracortical lesion volume	0.01	0.95	−0.20	0.21
Deep white matter lesion volume	0.06	0.73	−0.00	0.71

* indicates results are statistically significant adjusted for false-discovery rate.

**Figure 2:**
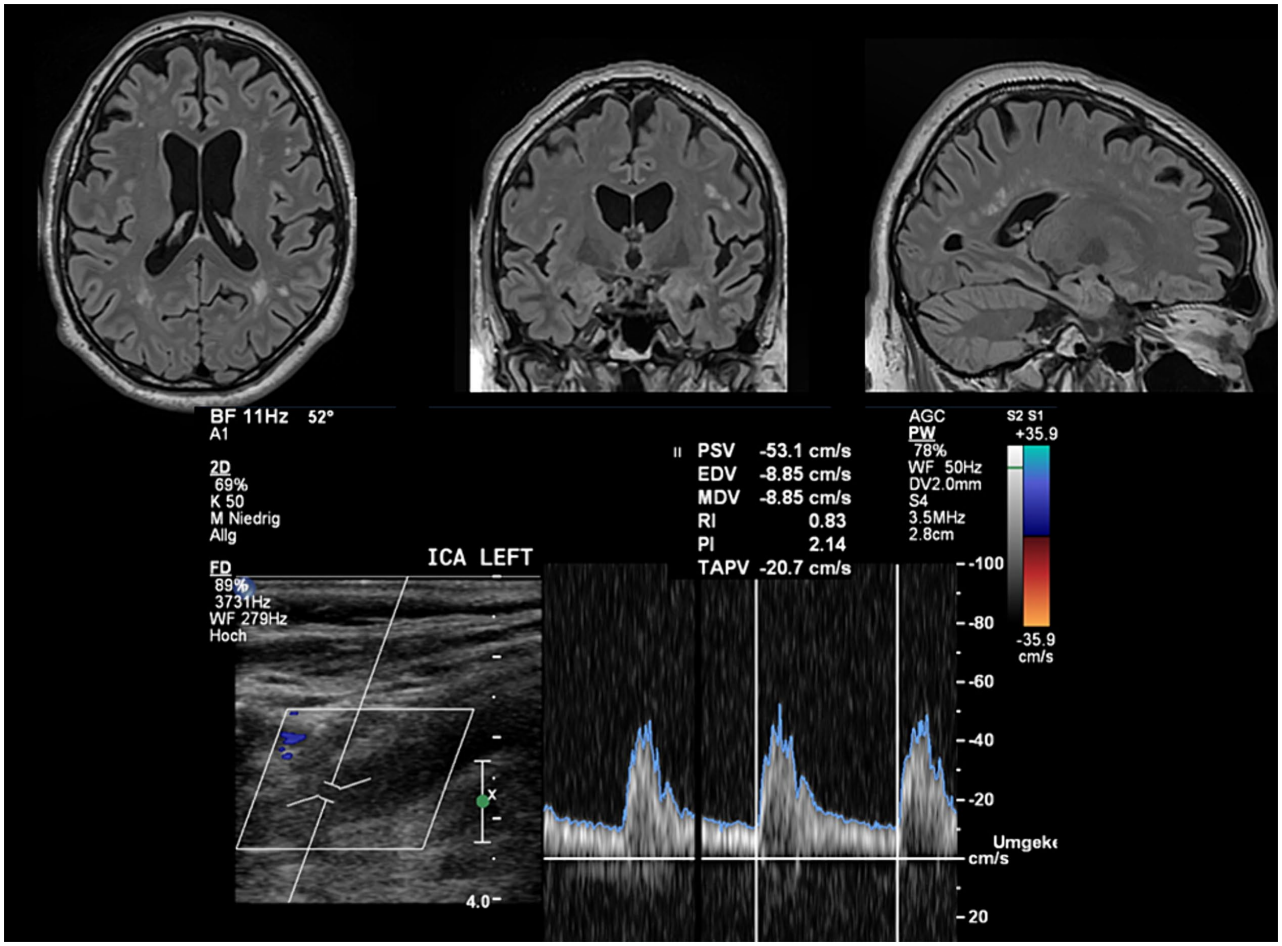
Exemplary case of a patient with high PI and white matter changes. FLAIR images depict a significant white matter lesion load and atrophy. The PI automatically estimated from the flow velocity envelope is enhanced in line with group results. The mean CERAD score of this patient was *z* = −1.38 indicating cognitive impairment lower than one standard deviation than the reference population.

**Figure 3:**
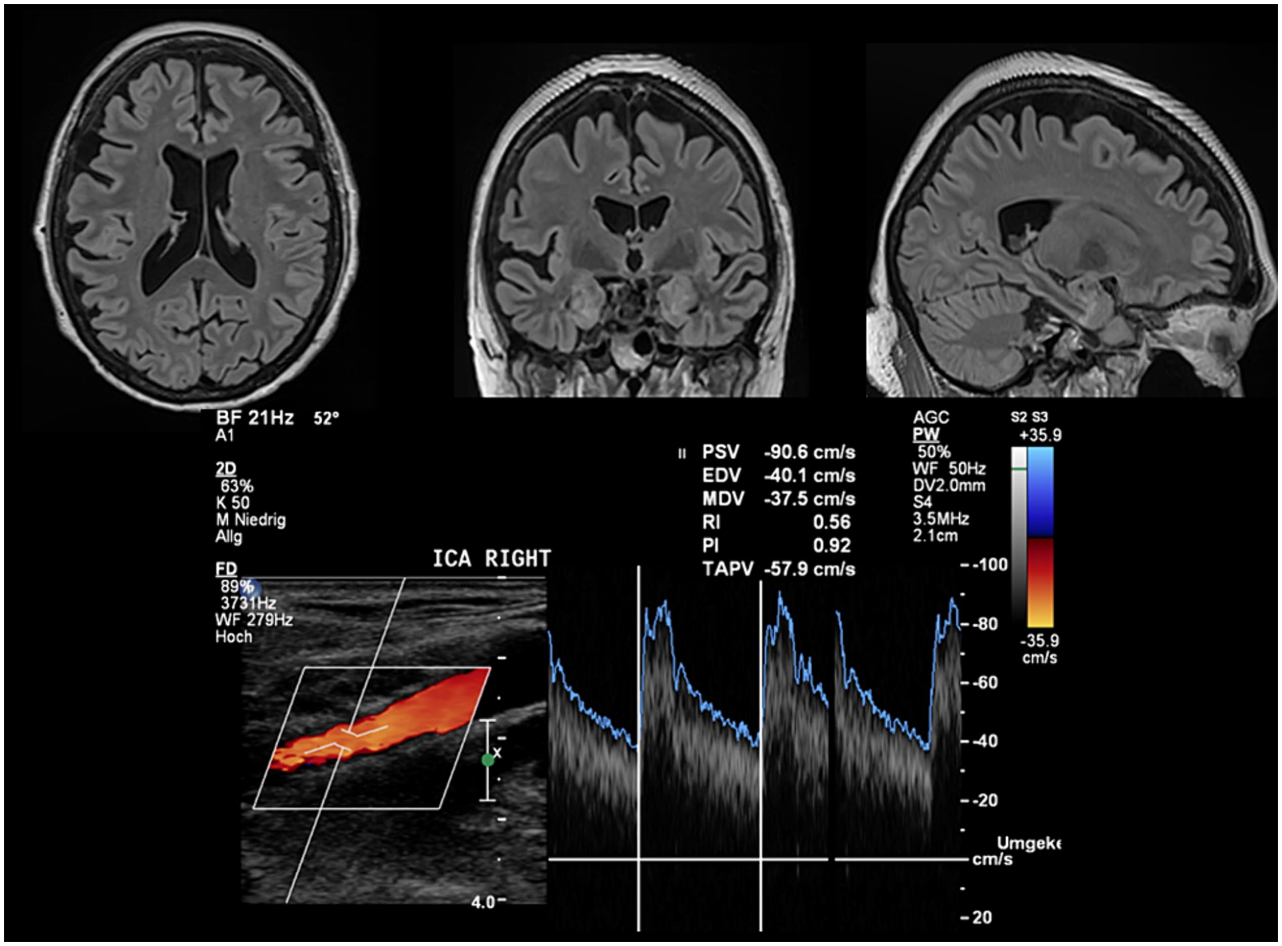
Exemplary case of a patient with low PI and absent white matter pathology. FLAIR images do neither indicate a relevant white matter lesion load nor atrophy. The PI automatically estimated from the flow velocity envelope is low in line with group results in patients without MRI abnormalities. The mean CERAD score of this patient was *z* = 0.46 indicating normal cognitive function compared to the reference population.

PI was significantly positively correlated with total (*τ* = 0.19, *p* = 0.012) and periventricular white matter lesion volume (*τ* = 0.21, *p* = 0.007). There was significant positive correlation of TAPV with white matter volume (*τ* = 0.20, *p* = 0.018), while PI was negatively correlated (*τ* = −0.14, *p* = 0.049). Exemplary cases of this correlation are illustrated in Figure 2 (high PI) and Figure 3 (low PI).

### 3.5 Multivariate analyses for the prediction of POD

The initial model, which included only the mean PI and constant, yielded an AIC of 65.4 and significantly enhanced the prediction of the dependent variable (*χ*^2^(1) = 7.5, *p* = 0.006). By adding the duration of surgery and age, the AIC was further reduced to 60.6, leading to a significant improvement in the model’s predictive power (*χ*^2^(3) = 16.4, *p* < 0.001). The odds of an episode of POD, as predicted by an increase in the PI adjusted for age and duration of surgery, were notably higher (OR_adj_ = 6.38 [95% CI: 1.77–23.03], *p* = 0.005) compared to the initial model. Neither baseline cognitive scores nor neuroimaging parameters contributed to improving the initial or the final model adjusted for age and duration of surgery, as indicated by the AIC.

## 4 Discussion

This study explored the hypothesis that quantitative CVDS could serve as surrogate markers for assessing the risk of POD in elderly patients undergoing elective spinal surgery. Our findings confirmed this hypothesis, revealing that even a one-point increase in the PI significantly elevates the risk of POD by approximately fivefold. Importantly, PI remained a strong predictor of POD even after adjusting for age and surgery duration, with the adjusted odds for POD increasing compared to the initial model. This indicates that PI is indeed an independent predictor of POD, a conclusion further supported by the fact that neither baseline cognitive abilities nor neuroimaging findings added any predictive value beyond what was achieved with PI alone. In contrast, CVDS measures of perfusion, i.e., TAPV, did not predict POD. This may be due to the lack of association between TAPV and neuroimaging findings, although this remains speculative. Moreover, ROC analyses suggest that while PI is effective in identifying patients at lower risk for POD. i.e. those without evidence of SVD and VCI, it is less effective in predicting those at higher risk. This could be because elevated PI might still be mitigated by other protective factors, while the absence of an increased PI effectively minimizes the risk for POD. This underscores the potential of the PI to be used to inform the risk for POD while also highlighting its limitations in offering a fully comprehensive risk assessment.

Multiple studies have investigated the feasibility and practicability of routine pre-operative CVDS, importantly of the ICA. A study spanning four years involved the noninvasive evaluation of carotid stenosis in over 2,000 patients undergoing cardiac surgery (24). The study emphasized the clinical relevance and applicability of ultrasound techniques in detecting cerebrovascular diseases prior to surgery, which potentially enhances surgical safety and planning. According to the Society for Vascular Surgery’s guidelines, preoperative carotid duplex ultrasound scanning is advocated due to its low cost, ease of use, and non-invasive nature (25). This approach is feasible and widely accepted for assessing carotid stenosis in patients undergoing various surgical procedures. Another study investigated whether results from ICA CVDS are comparable between expert users and untrained users. This study suggested that non-specialists could effectively use ultrasound in clinical settings, implying potential for lab technicians under proper training (26). This further extends the utility of this readily and broadly available technique to inform the risk assessment for POD in addition to other well-established parameters such as age and duration of surgery.

Several studies have investigated plausible correlates for the identified association between ICA PI, the presence of white matter lesions, and inferior cognitive function. The extent of white matter lesions was shown to be associated with arterial remodeling, leading to increased arterial stiffness and impaired arterial blood flow dynamics (27). Further evidence links the presence of white matter lesions to small vessel disease, which increases peripheral resistance and leads to increased ICA PI (28). Another study reported indirect evidence for this association and found that the prevalence and severity of white matter lesions were associated with several vascular risk factors, including hypertension, diabetes, and smoking. These factors also contribute to the pathophysiology of small vessel disease, suggesting shared pathways (29). Finally, there is direct evidence that PI, but not blood flow as measured by TAPV, is affected by cerebral small vessel disease, underpinning our finding that increased PI, but not TAPV, is associated with vascular cognitive impairment (22). Insonation of intracranial arteries was hindered by an unexpectedly high incidence of inadequate temporal bone windows. The success rate of only about 50% in our study appears to contradict previous findings, even in older patients (30). However, we applied stricter criteria, requiring the identification of major vessel segments rather than just the isolated depiction of vessels. That being said, complete visualization of intracranial arteries was also achieved in only about 50% of cases in the study by Hoksbergen et al., which aligns with our findings.

Identifying patients at risk for POD plays a pivotal role in mitigating the effects of postoperative neurocognitive disorders, as there is little evidence for the treatment of manifest POD (31). The evidence for multicomponent interventions for the prevention of POD, however, is profound (32). Thus, multiple risk prediction models have been developed, the accuracy of which might benefit from preoperative CVDS since they rarely include preoperative cognitive assessments or neuroimaging due to their resource intensity (33). This approach is particularly intriguing in cardiac surgery since vascular ultrasound is often done as part of the evaluation before on-pump coronary bypass surgery.

The detection of small vessel disease using preoperative CVDS could play a crucial role in minimizing the risk for POD through optimized blood pressure management during surgery. Small vessel disease is associated with alterations in cerebral blood flow and vascular reactivity, which can be exacerbated by intraoperative blood pressure fluctuations. Preoperative identification of microvascular changes allows anesthesiologists to tailor blood pressure management strategies to maintain stable cerebral perfusion pressure, thereby preventing episodes of hypo- or hyperperfusion that might lead to critical blood flow, a known risk factor for POD. One study reported that fluctuation of the CPP by 10 mmHg is associated with a twofold increased risk for POD (34).

The study was terminated early due to the triage of elective surgical procedures during the SARS-CoV-2 pandemic, which is a limitation of the findings. As a result, the smaller dataset may have affected the investigation of secondary endpoints, and marginally significant findings might have statistically significant if the study had continued as planned. Nonetheless, even small to moderate correlations were identified rendering findings a contribution to the understanding of POD pathophysiology. The prone position, frequently utilized in spine surgery, is another potential limitation of this study, which may impede generalizability. It has been shown that prone position my increase intracranial pressure (ICP), which can aggravate preexisting high vascular resistance (35). This hemodynamic effect may impair cerebral autoregulation, thereby contributing to the higher rates of POD observed in these patients. These effects may be less pronounced in the supine position, where ICP increases are typically more moderate, potentially leading to different postoperative outcomes. Finally, results might differently affect cardiac surgery, particularly during cardiopulmonary bypass (CPB), which introduces unique hemodynamic challenges. The laminar flow characteristic of CPB lacks the systolic peaks seen in normal pulsatile flow, which may impair cerebral perfusion pressure (CPP) more severely in patients with increased vascular resistance, as indicated by an elevated pulsatility index (PI) (36). This impairment could exacerbate hypoperfusion, particularly in patients with small vessel disease (SVD), leading to more pronounced neurocognitive decline or postoperative delirium (POD). The high incidence of POD in cardiac surgery, reported to be as high as 70%, could be partly explained by these factors. Therapeutic approaches to manage fluctuations in CPP, such as optimizing mean arterial pressure and ensuring adequate cerebral perfusion during surgery, should be considered to mitigate these risks.

In conclusion, this study confirms that quantitative CVDS can serve as a surrogate marker for POD in elderly patients undergoing elective spinal surgery. A key finding is that increased PI substantially raises the risk of POD. The potential of CVDS as a routine screening tool to estimate the risk for POD is supported by its accessibility, ease of use, and efficiency compared to more resource-intensive methods like comprehensive neurocognitive testing and MRI. This method can enhance surgical safety and planning by informing risk assessment for POD, for both of which CVDS provides surrogate markers. The identification of potential SVD may inform optimized management of blood pressure during surgery to maintain stable cerebral perfusion pressure and minimize cognitive consequences of otherwise beneficial surgery in elderly patients. Prospective studies should investigate this intriguing perspective in risk prediction models, including age.

## Data Availability

The raw data supporting the conclusions of this article will be made available by the authors, without undue reservation.
